# Administration of zinc to preterm infants with hypozincemia does not reduce serum copper concentrations in most cases: a single-center retrospective observational study

**DOI:** 10.1186/s40780-021-00229-4

**Published:** 2021-12-02

**Authors:** Toshikazu Ito, Kazuya Uenoyama, Kazuhiro Kobayashi, Mikio Kakumoto, Hiroshi Mizumoto, Toshiya Katsura, Masahide Onoue

**Affiliations:** 1 Department of Pharmacy, Kitano Hospital, Tazuke Kofukai Medical Research Institute. 2-4-20, Ogimachi, Kita-ku, Osaka, 530-8480 Japan; 2 Laboratory of Clinical Pharmaceutics and Therapeutics, College of Pharmaceutical Sciences, Ritsumeikan University, 1-1-1, Noji-higashi, Kusatsu, Shiga 525-8577 Japan; 3 Laboratory of Clinical Pharmacy, College of Pharmaceutical Sciences, Ritsumeikan University, 1-1-1, Noji-higashi, Kusatsu, Shiga 525-8577 Japan; 4 Department of Pediatrics, Kitano Hospital, Tazuke Kofukai Medical Research Institute, 2-4-20, Ogimachi, Kita-ku, Osaka, 530-8480 Japan

**Keywords:** Preterm infants, Zinc, Copper, Serum concentration, Hypozincemia

## Abstract

**Background:**

Zinc is an essential trace element involved in various physiological functions. In Japan, zinc acetate dihydrate is administered to neonates and infants with hypozincemia. Since serum copper concentrations are reduced by the administration of zinc, we retrospectively investigated changes in serum zinc and copper concentrations in preterm infants with hypozincemia receiving zinc acetate dihydrate.

**Methods:**

Sixty-three preterm infants were included in the present study. Serum zinc and copper concentrations, doses, and other clinical characteristics were retrieved from electronic medical records.

**Results:**

The medians and interquartile ranges of the dosage and duration of zinc acetate dihydrate were 2.1 (1.8–2.5) mg/kg/day and 12.0 (10.0–13.0) days, respectively. Its administration increased serum zinc concentrations in 39 patients (61.9%) and to more than 70 μg/dL in 16 patients (25.4%). The group with a serum zinc concentration of 70 μg/dL or higher after administration had a significantly higher zinc dose of 2.5 mg/kg/day than the group with a serum zinc concentration of less than 70 μg/dL. Serum copper concentrations did not decrease in 44 patients (69.8%). In the group with a decreased serum copper concentration, postmenstrual age and body weight were significantly lower, while serum zinc concentrations were significantly higher at the start of administration.

**Conclusion:**

The present results showed that when zinc acetate dihydrate was administered to preterm infants with hypozincemia, it was possible to increase serum zinc concentrations without decreasing serum copper concentrations in many cases. However, caution may be required when administering zinc to preterm infants with a lower postmenstrual age or milder hypozincemia because serum copper concentrations may decrease.

## Background

Zinc is an essential trace element involved in various physiological functions, such as taste sensations, immune functions, skin metabolic functions, and skeletal development, and is required for the in vivo activation of more than 300 enzymes [1].

Preterm infants are prone to zinc deficiency due to low stores of zinc during the prenatal period and an inadequate zinc intake. Fetal zinc stores rapidly increase after 30 weeks of gestation [2], while zinc concentrations in breast milk gradually decrease postnatally [3]. Zinc deficiency in neonates and infants is associated with dermatitis, growth failure, infection, and autism spectrum disorde r[4, 5]. In Japan, zinc acetate dihydrate has been approved as a therapeutic agent for hypozincemia and is also administered to neonates and infants with hypozincemia as off-label use [6].

A decrease in copper concentrations has been reported as adverse effects of zinc administration [7]. The intestinal absorption of zinc and copper is performed by transporters in small intestinal epithelial cells. Zrt−/Irt-like protein 4 and zinc transporter 1 play roles in zinc absorption in the apical and basolateral membranes [8], respectively, while copper absorption involves copper transporter 1 (CTR1) and copper-transporting ATPase 1 (ATP7A) in the apical and basolateral membranes [9], respectively. Furthermore, zinc and copper induce the expression of metallothionein, which contributes to their homeostasis. Copper has a higher affinity to metallothionein than zinc [10]. Therefore, zinc decreases serum copper concentrations by inducing the expression of metallothionein, which binds to copper and inhibits absorption.

In our Neonatal Intensive Care Unit (NICU), blood is collected approximately every two weeks as a regular blood test for medical evaluations. Based on the findings of blood sampling, the administration of zinc acetate dihydrate is generally initiated for neonates and infants with hypozincemia whose serum zinc concentration is lower than 70 μg/dL [11]. The administration of zinc acetate dihydrate is initiated at a dose of approximately 2 mg/kg/day, and this dose is adjusted based on the results of regular blood tests. Copper deficiency, one of the side effects associated with zinc administration, may lead to anemia, pancytopenia, and neuropathy [12]. Although the administration of zinc increases the risk of copper deficiency, there is currently no information on changes in serum zinc or copper concentrations in preterm infants after its administration. Therefore, to evaluate efficacy and safety in the early post-dose period, we retrospectively investigated serum zinc and copper concentrations in preterm infants administered zinc acetate dihydrate for hypozincemia during NICU hospitalization.

## Materials and methods

### Patients

**Fig. 1:**
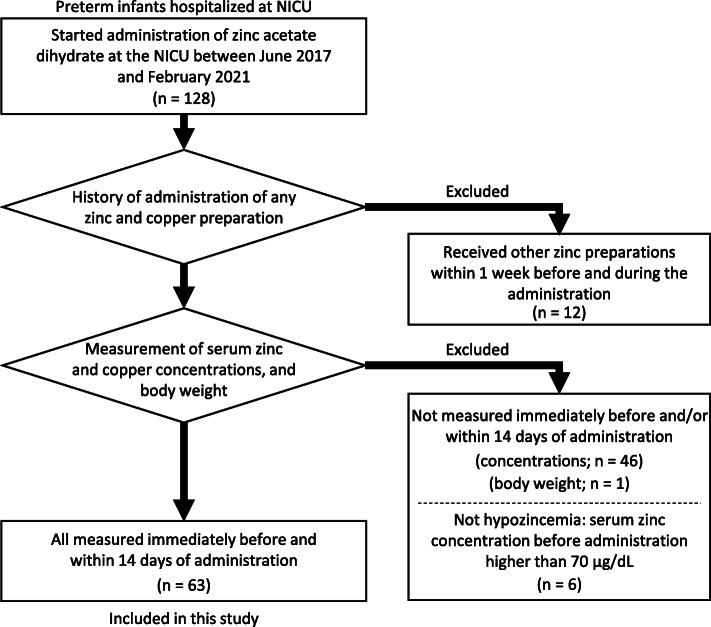
Flowchart of study participants

Between June 2017 and February 2021, 128 preterm infants under 37 weeks of gestation began receiving zinc acetate dihydrate during their admission to the NICU. Twelve infants who received other zinc preparations within 1 week before and during the administration of zinc acetate dihydrate were excluded. In all infants, zinc was only orally administered as zinc acetate dihydrate, and none of the infants received copper preparations. Forty-six infants with no data on serum zinc or copper concentrations immediately before and/or within 14 days of administration, 6 with serum zinc concentrations of 70 μg/dL or higher before the start of treatment, and 1 whose body weight was not measured during administration were excluded (Fig. 1).

### Data collection

Data on gestational age (GA), body weight and height, the dose and duration of the zinc acetate dihydrate treatment, and serum zinc and copper concentrations immediately prior to the start of the zinc acetate dihydrate administration and in the first clinical examination after its administration were retrospectively retrieved from electronic medical records. The dose of each case was calculated for each change in body weight during the administration period, and the average value was used for the analysis.

### Serum zinc and copper concentration measurements

Measurements of serum zinc and copper concentrations were conducted as routine clinical practice. Serum zinc concentrations were assessed using a direct colorimetric assay based on the 5-Br-PAPS method by JCM-BM6070 BioMajesty (JEOL Ltd., Tokyo). ACCURAS AUTO Zn (Shino-Test, Tokyo) was used as a reagent.

Serum copper concentrations were assessed using a direct colorimetric assay based on the 3,5-DiBr-PAESA method with JCM-BM6070 BioMajesty (JEOL Ltd., Tokyo). QUICK AUTO NEO Cu (Shino-Test, Tokyo) was used as a reagent.

### Statistical analysis

Data were expressed as medians (interquartile ranges). The Shapiro-Wilk normality test was used to test for normality. In the test of two matched groups, the paired *t*-test for normality and the Wilcoxon signed-rank test for non-normality were used. In the test of two independent groups, the Student’s *t*-test for normality and equal variance, the Welch’s test for normality and unequal variance, and the Mann-Whitney U test for non-normality were used. The significance of differences was set to *P* < 0.05. All statistical analyses were performed with EZR (Saitama Medical Center, Jichi Medical University, Saitama, Japan), which is a graphical user interface for R (The R Foundation for Statistical Computing, Vienna, Austria). More precisely, it is a modified version of R commander designed to add statistical functions frequently used in biostatistics.

## Results

### Patient background

In the present study, 63 patients fulfilled the inclusion criteria, and their demographics and clinical characteristics are summarized in Table 1. Males accounted for 52.4% (*n* = 33) of all cases, and median GA and postmenstrual age (PMA) were 32.0 (30.3–33.9) and 35.6 (34.6–36.9) weeks, respectively. The median dosage and duration of administration of zinc acetate dihydrate until the first clinical examination were 2.1 (1.8–2.5) mg/kg/day and 12.0 (10.0–13.0) days, respectively. Median serum zinc and copper concentrations prior to administration were 58.0 (54.5–64.0) and 42.0 (32.0–47.5) μg/dL, respectively. Although serum copper concentrations were less than 40 μg/dL in some patients during this period, none exhibited the symptoms of copper deficiency, such as neutropenia, or required the administration of copper. In addition, during the administration period, the exacerbation of gastrointestinal symptoms, such as vomiting and diarrhea, was not observed as a side effect of zinc acetate dihydrate in any patients.

**Table 1:** Clinical characteristics

	n	Median	Range (Interquartile range)
Sex			
Male	33 (52.4%)		
Female	30 (47.6%)		
GA (week)		32.0	25.0–36.1 (30.3–33.9)
Birth weight (g)		1556.0	624.0–2550.0 (1210.5–1782.5)
Dose (mg/kg/day)		2.1	1.0–3.8 (1.8–2.5)
Administration period (day)		12.0	4.0–14.0 (10.0–13.0)
At the start of administration			
PNA (day)		26.0	9.0–72.0 (15.0–31.5)
PMA (week)		35.6	31.4–39.6 (34.6–37.0)
Body weight (g)		1948.0	866.0–2686.0 (1752.0–2212.5)
Serum zinc concentration (μg/dL)		58.0	40.0–69.0 (54.5–64.0)
Serum copper concentration (μg/dL)		42.0	26.0–86.0 (32.0–47.5)

GA, gestational age; PNA, postnatal age; PMA, postmenstrual age

### Effects of the administration of zinc acetate dihydrate on serum zinc concentrations

**Fig. 2:**
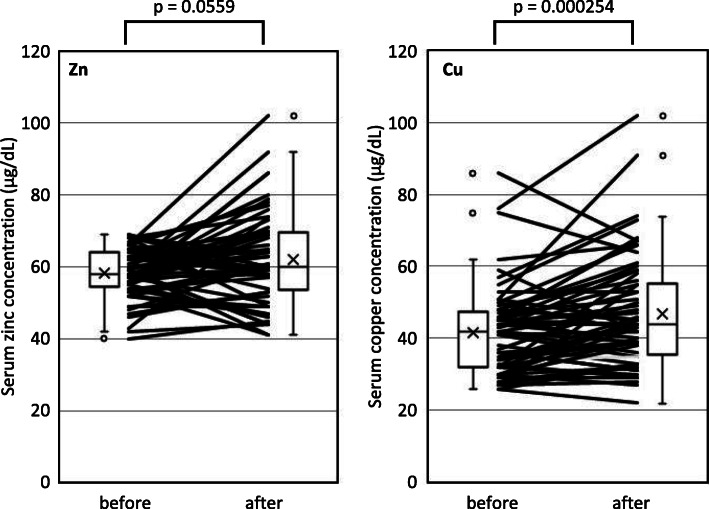
Changes in serum zinc and concentrations before and after the administration of zinc acetate dihydrate. The Wilcoxon signed-rank test and paired *t*-test were used to analyze serum zinc and copper concentrations, respectively.

Serum zinc concentrations after the administration of zinc acetate dihydrate increased and did not increase in 61.9% (*n* = 39) and 38.1% of patients (*n* = 24), respectively. The administration of zinc acetate dihydrate slightly increased the serum zinc concentration from 58.0 (54.5–64.0) to 60.0 (53.5–69.5) μg/dL (*p* = 0.0559) (Fig. 2).

The administration of zinc acetate dihydrate increased serum zinc concentrations to higher than 70 μg/dL in 16 patients. Tables 2 and 3 show a comparison between the group in which serum zinc concentrations increased to 70 μg/dL or higher and the group in which they did not reach 70 μg/dL. Average doses were 2.5 (2.2–2.9) and 2.0 (1.5–2.3) mg/kg/day, respectively, which was significantly higher in the former group (*p* = 0.000456). In addition, serum copper concentrations before the start of administration of zinc acetate dihydrate were 47.0 (42.5–53.5) and 36.0 (32.0–45.0) μg/dL, respectively, and were significantly higher in the former group (*p* = 0.0174). No significant differences were observed in other items.

**Table 2:** Clinical characteristics by serum zinc concentration after zinc administration

	≧ 70 μg/dL (*n* = 16)		< 70 μg/dL (*n* = 47)		
	Median	Range (Interquartile range)	Median	Range (Interquartile range)	*p*
GA (week)	31.4	25.0–36.1 (29.8–33.0)	32.6	26.4–36.1 (30.4–34.0)	0.252
Birth weight (g)	1315.5	624.0–2300.0 (1175.8–1700.5)	1578.0	782.0–2550.0 (1264.0–1782.5)	0.272
Dose (mg/kg/day)	2.5	1.7–3.8 (2.2–2.9)	2.0	1.0–3.3 (1.5–2.3)	0.000456
Administration period (day)	12.0	6.0–14.0 (10.0–13.0)	13.0	4.0–14.0 (9.0–13.0)	0.513
At the start of administration					
PNA (day)	29.0	14.0–71.0 (25.0–35.3)	22.0	9.0–72.0 (15.0–30.5)	0.0542
PMA (week)	35.8	31.4–39.6 (34.4–37.1)	35.4	32.6–38.3 (34.6–36.8)	0.953
Body weight (g)	2035.5	866.0–2617.0 (1659.0–2223.0)	1946.0	1275.0–2686.0 (1763.0–2212.5)	0.0778
Serum zinc concentration (μg/dL)	58.5	43.0–68.0 (56.0–64.5)	58.0	40.0–69.0 (53.0–63.5)	0.472
Serum copper concentration (μg/dL)	47.0	27.0–86.0 (42.5–53.5)	36.0	26.0–76.0 (32.0–45.0)	0.0174

The Student’s *t*-test was used to analyze GA, Birth weight, Dose and Serum zinc concentration. The Welch’s test was used to analyze PMA and Body weight. The Mann-Whitney U test was performed for other variables. GA, gestational age; PNA, postnatal age; PMA, postmenstrual age

**Table 3:** Other characteristics by serum zinc concentration after zinc administration

	≧ 70 μg/dL (*n* = 16)	< 70 μg/dL (*n* = 47)	*p*
Sex			
Male	9	35	0.778
Female	7	12	
Enteral nutrition			
Breast milk	7	18	0.899
Infant formula	1	3	
Breast milk + Infant formula	8	26	

The Fisher’s exact test was used for statistical analyze

### Effects of the administration of zinc acetate dihydrate on serum copper concentrations

Serum copper concentrations after the administration of zinc acetate dihydrate decreased and did not decrease in 30.2% (*n* = 19) and 69.8% of patients (*n* = 44), respectively. The administration of zinc acetate dihydrate significantly increased the serum copper concentration from 42.0 (32.0–47.5) to 44.0 (35.5–55.5) μg/dL (*p* = 0.000254) (Fig. 2).

Tables 4 and 5 show a comparison between the group in which serum copper concentrations decreased and the group in which they did not decrease. PMA were 34.9 (33.6–35.9) and 36.0 (35.1–37.0) weeks, respectively, which was significantly lower in the former group (*p* = 0.0235). Body weights at the start of administration were 1740.0 (1610.0–2052.5) g and 1979.5 (1831.0–2257.3) g, respectively, which was significantly lower in the former group (*p* = 0.0236). In addition, serum zinc concentrations before the start of administration were 62.0 (57.5–66.0) and 57.0 (53.0–63.0) μg/dL, respectively, which was significantly higher in the former group (*p* = 0.024). No significant differences were observed in other items.

**Table 4:** Clinical characteristics by changes in serum copper concentration after zinc administration

	decrease (*n* = 19)		Not decrease (*n* = 44)		
	Median	Range (Interquartile range)	Median	Range (Interquartile range)	*p*
GA (week)	31.1	25.0–36.1 (29.5–33.0)	32.2	26.4–36.1 (30.6–34.0)	0.193
Birth weight (g)	1264.0	624.0–2312.0 (1131.5–1802.0)	1576.5	782.0–2550.0 (1282.5–1780.8)	0.197
Dose (mg/kg/day)	2.0	1.1–3.4 (1.5–2.5)	2.1	1.0–3.8 (1.8–2.5)	0.517
Administration period (day)	13.0	4.0–14.0 (9.0–13.0)	12.0	5.0–14.0 (10.0–13.0)	0.724
At the start of administration					
PNA (day)	26.0	9.0–45.0 (16.5–29.0)	25.5	12.0–72.0 (15.0–32.8)	0.881
PMA (week)	34.9	31.4–39.6 (33.6–35.9)	36.0	32.9–39.3 (35.1–37.0)	0.0235
Body weight (g)	1740.0	866.0–2686.0 (1610.0–2052.5)	1979.5	1382.0–2620.0 (1831.0–2257.3)	0.0236
Serum zinc concentration (μg/dL)	62.0	52.0–68.0 (57.5–66.0)	57.0	40.0–69.0 (53.0–63.0)	0.024
Serum copper concentration (μg/dL)	42.0	26.0–86.0 (36.0–49.0)	39.0	26.0–76.0 (31.5–46.3)	0.222

The Student’s *t*-test was used to analyze GA, Birth weight, Dose and Serum zinc concentration. The Welch’s test was used to analyze PMA and Body weight. The Mann-Whitney U test was performed for other variables. GA, gestational age; PNA, postnatal age; PMA, postmenstrual age

**Table 5:** Other characteristics by changes in serum copper concentration after zinc administration

	decrease (*n* = 19)	Not decrease (*n* = 44)	*p*
Sex			
Male	10	23	1.0
Female	9	21	
Enteral nutrition			
Breast milk	10	15	0.245
Infant formula	0	4	
Breast milk + Infant formula	9	25	
Serum zinc concentration after administration			
≧ 70 μg/dL	7	9	0.212
< 70 μg/dL	12	35	

The Fisher’s exact test was used for statistical analyze

## Discussion

The administration of zinc acetate dihydrate was previously predicted to reduce serum copper concentrations [7]; however, in the present study on preterm infants, there were more cases in which serum copper concentrations did not decrease. Since significant differences were observed in PMA and serum zinc concentrations before the start of administration between the groups with and without decreased serum copper concentrations, we proposed the following three mechanisms.

The first mechanism involves fluctuations in copper absorption in the small intestine during development. The absorption efficiency of copper in the small intestine of rats was previously shown to fluctuate during development and was higher 10 days after birth than 20 days after birth [13]. Although the relationship between development and copper absorption efficiency in the small intestine of humans currently remains unknown, copper absorption efficiency may also fluctuate in infants. Therefore, further studies are required to clarify the relationship between development (e.g., PNA and PMA) and the efficiency of copper absorption (e.g., expression levels of CTR1 and ATP7A).

The second mechanism involves the effects of serum zinc concentrations at the start of administration. The induction of metallothionein expression is dependent on zinc concentrations [14]. In the present study, zinc acetate dihydrate was administered to patients with hypozincemia. Since serum zinc concentrations at the start of administration were low, they may not have increased to levels that induce the expression of metallothionein.

The third mechanism involves fluctuations in the expression of metallothionein in the small intestine during development. Nishimura et al. investigated the postnatal developmental stage and expression of metallothionein in the small intestine of rats and found no marked changes in the expression level of metallothionein until 18 days after birth; however, its expression peaked 27 days after birth and then gradually decreased [15]. Although the relationship between development and the expression of metallothionein in the small intestine of humans is currently unclear, serum copper concentrations may not have decreased after the administration of zinc because the administration of zinc acetate dihydrate was initiated when the expression level of metallothionein was low.

In the gastrointestinal absorption of zinc, copper has been reported to inhibit zinc influx in a concentration-dependent manner in the intact rat intestine [16]. On the other hand, in the present study, a higher serum copper concentration at the start of administration was associated with a higher serum zinc concentration. This may be attributed to the interaction between serum copper and zinc concentrations in the present study differing from the previously reported mechanism for the interaction between copper and zinc in the intestinal tract during the absorption process. Although the relationship between elevated serum zinc and copper concentrations currently remains unclear, serum copper concentrations at the start of treatment had a significant effect on the outcome of treatment for hypozincemia in the present study.

In the present study, serum zinc concentrations were slightly increased by the administration of zinc acetate dihydrate. The recommended dose of zinc intake in preterm infants is reported to be 1–2 mg/kg/day [17]. However, in the present study, the median average dose during administration was 2.5 mg/kg/day in the group in which the serum zinc concentration increased to 70 μg/dL or higher, whereas it was 2.0 mg/kg/day in the group in which the serum zinc concentration did not reach 70 μg/dL. Therefore, the dose of 2.0 mg/kg/day may not be sufficient to treat hypozincemia in preterm infants in a shorter period of time.

There are two limitations in the present study that need to be addressed. The number of cases was small and the developmental effects of zinc and copper absorption in preterm human infants currently remain unknown. We plan to increase the number of cases and investigate the factors that were herein found to increase and decrease in serum zinc and copper concentrations after the administration of zinc acetate dihydrate using a multivariate analysis. In addition, PMA was identified as a significant factor contributing to increases and decreases in serum copper concentrations after the administration of zinc acetate dihydrate, which indicates that the absorption efficiency of copper fluctuates in the process of development. Future studies are needed to evaluate the absorption and excretion rates of copper over time in preterm human infants and to assess changes in copper absorption efficiency due to development.

## Conclusion

In the present study, the administration of zinc acetate dihydrate to preterm infants with hypozincemia was safe and did not reduce serum copper concentrations in many cases. However, a decrease in serum copper concentrations was observed in some cases. Therefore, to administer zinc preparations more safely as a treatment for hypozincemia in preterm infants, further studies on the factors that reduce serum copper concentrations are needed. In addition, to more effectively treat hypozincemia in preterm infants, future research that investigates the effects of serum copper concentrations on elevated serum zinc concentrations and the need for higher zinc doses is required.

## Data Availability

The datasets used and/or analyzed during the present study are available from the corresponding author upon reasonable request.

## Abbreviations

NICU: Neonatal Intensive Care Unit
GA: gestational age
PNA: postnatal age
PMA: postmenstrual age
CTR1: copper transporter 1
ATP7A: copper-transporting ATPase 1
